# HI-PLUS: design of a cluster-randomized trial evaluating structured case management with telemonitoring to improve quality of life in heart failure

**DOI:** 10.1007/s00392-026-02904-8

**Published:** 2026-04-27

**Authors:** Martha Schutzmeier, Viktoria Rücker, Jonas Widmann, Peer Papior, Lisa Kimmelmann, Fabian Kerwagen, Lorenz Heil, Helena Manger, Andrea Szczesny, Yannick Maaser, Bettina Zippel-Schultz, Thomas Helms, Peter U Heuschmann, Stefan Störk

**Affiliations:** 1 Institute for Clinical Epidemiology and Biometry, University of Würzburg, Würzburg, Germany; 2 Dept. Clinical Research & Epidemiology, Comprehensive Heart Failure Center Würzburg, and Dept. Internal Medicine I, University Hospital Würzburg, Würzburg, Germany; 3 Department of Business Management, University of Würzburg, Würzburg, Germany; 4 German Foundation for the Chronically Ill, Fürth, Germany; 5 Clinical Trial Center, University Hospital Würzburg, Würzburg, Germany; 6 Institute of Medical Data Science, University Hospital Würzburg, Würzburg, Germany

**Keywords:** Heart failure, Quality of life, Cluster randomized trial, Care and case management, Telemonitoring

## Abstract

**Introduction:**

Heart failure (HF) remains a leading cause of hospitalization and death in Germany, significantly impairing patients’ quality of life (QoL) despite advances in therapy. Structured, multidisciplinary care programs may improve long-term outcomes. HI-PLUS is a pragmatic trial investigating whether a complex intervention can improve QoL in HF patients. We describe the trial design and pilot feasibility study findings.

**Methods and results:**

HI-PLUS is a cluster-randomized, parallel-arm controlled trial (DRKS00031997) targeting 56 clusters, each comprising a cardiology practice and up to five general practitioners (GPs), targeting 1350 patients. The control arm receives guideline-recommended care. The intervention adds five elements: (a) care supported by specially trained non-physician staff HF qualified (HF-MPA); (b) accredited HF-MPA training curriculum certified by the German Society of Cardiology; (c) eHealth platform for telemonitoring and symptom reporting; (d) portable telemedical devices, if needed; and (e) enhanced communication between cardiologists and GPs facilitated by HF-MPAs and the eHealth platform. The primary endpoint is change in HF-specific QoL after 12 months, measured by the Kansas City Cardiomyopathy Questionnaire Overall Summary Score (KCCQ-OSS). A pilot feasibility study that was conducted prior to commencement of patient recruitment confirmed the feasibility and acceptability of the proposed trial procedures.

**Conclusion:**

The HI-PLUS trial addresses recognized gaps in HF care. If effective, it could serve as a scalable model for integrated HF care throughout Germany.

**Graphical Abstract:**

**Figure:**
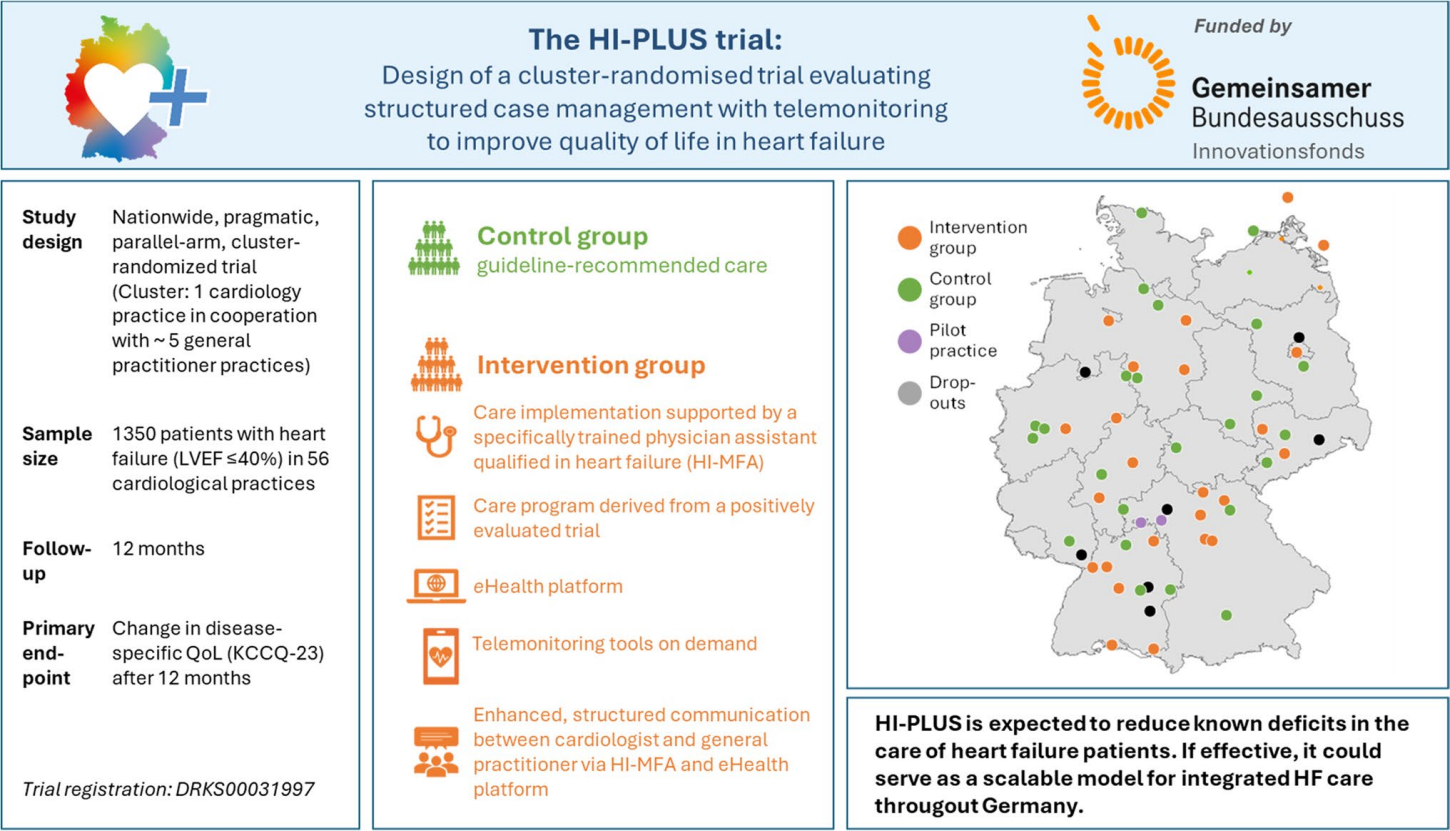

**Supplementary Information:**

The online version contains supplementary material available at 10.1007/s00392-026-02904-8.

## Introduction

Although the incidence of heart failure (HF) has remained relatively stable, the prevalence continues to rise as advances in evidence-based therapies and care prolong survival [1]. Yet, recurrent hospitalizations remain very frequent, placing a heavy burden on healthcare systems and diminishing patients’ quality of life (QoL) [2]. Accordingly, preserving or improving health-related QoL is thus a core therapeutic aim in chronic HF care, alongside traditional clinical outcomes [3, 4].

Contemporary European guidelines recommend that patients with HF participate in a multidisciplinary management program [2]. Case-management and multidisciplinary approaches, particularly those led by specialist nurses, can reduce mortality and HF readmission rates [5]. Such programs can be clinic-based, home-based, or hybrid, and may incorporate telemonitoring. To be effective, they should be patient-centered, address comorbid conditions, enhance self-management skills, and be tailored to the resources of the healthcare system and to individual patient needs [5, 6]. Telemonitoring, for instance, has been shown to sustain care quality, enable timely interventions, and reduce the need for travel and in-person visits [7], in particular for patients who live far away from their cardiologist [8].

In Germany, HF outpatient care is primarily delivered by general practitioners (GPs), although outpatient cardiologists are also a component of the patient care pathway [3]. Currently, there is no comprehensive nationwide disease management program (DMP) to ensure standardized and coordinated care [3]. This fragmentation contributes to suboptimal implementation of guideline-recommended treatment, including underuse of evidence-based pharmacotherapy and limited communication across care sectors. Importantly, providing the time-intensive, individualized care that many HF patients require remains a challenge in outpatient practice [2]. Prior initiatives—such as the Interdisciplinary Network for Heart Failure (INH) study, which evaluated the impact of a nurse-led disease management program on mortality and morbidity in patients with heart failure [9, 10], and the Telemedical Interventional Management in Heart Failure II (TIM-HF2) trial, which assessed the effect of a structured remote patient management system on unplanned hospital admissions for cardiovascular reasons and mortality [11, 12]—demonstrated favorable effects yet underscored the ongoing need for scalable, patient-centered, evidence-based HF care across Germany.

In 2018, the Federal Joint Committee (G-BA) of Germany proposed a DMP for HF patients (DMP-HI) [13]. In 2021, the Institute for Quality and Efficiency in Health Care (IQWiG) subsequently identified areas for updating and recommended additional measures [14]. The IQWiG also provided an evidence mapping on structured support interventions for HF monitoring, intended to inform targeted decisions on potentially useful additions to the DMP-HI [15]. In 2024, the G-BA updated the DMP-HI to integrate telemonitoring [13], which is reimbursed by statutory health insurance since January 2022 for patients with NYHA functional class II or III and guideline-based treatment [16].

### Aim of the trial

We report the design and results of the pilot feasibility study of the ongoing HI-PLUS trial in Germany, which tests whether a structured, evidence-based care and case-management program, augmented by an eHealth platform, improves health-related QoL compared with usual care.

## Methods

### Trial design and randomization

The HI-PLUS trial is a pragmatic, parallel-arm, cluster-randomized study being conducted between June 2023 and June 2026. Cardiology practices (CPs) serve as the units of randomization because the intervention is delivered at the practice level and involves coordination with affiliated general practitioners (GPs). Clusters are generated by a single CP and up to five cooperating GPs and are assigned in a 1:1 ratio to either the intervention or control arm using stratified block randomization generated by PASS 2020 software. Stratification factors include the presence of a HF medical practice assistant (HF-MPA) at baseline. This design permits comparison of the complex intervention with usual care while accommodating the realities of practice-level implementation and minimizing contamination. Further information about the trial organization is listed in Supplementary Table S1.

### Setting and recruitment

CPs were recruited through professional associations, specifically the German Association of Registered Cardiologists (BNK). The target was 56 CPs (28 per group) working with approximately 280 GPs. Eligibility required CPs to consent to random allocation, deliver care per assignment, train 1–3 non-physician staff members if randomized to the intervention, and coordinate the network together with approximately 2 to 7 GPs (mean 5). CPs in the control arm that already employed a HF-MPA can participate provided a formal HF care pathway has not yet been implemented. A care pathway is considered implemented if the HF-MPA actively acts as an interface in the following manner between the cardiologist, the GP, the hospital, and the patient: (a) records and assesses HF symptoms, liaises with the supervising cardiologist, and ensures implementation of the recommendations is implemented; and/or (b) monitors the therapy (via telephone or telemonitoring) and provides outpatient coaching as required. The project team surveys CPs quarterly to ascertain whether a HF care pathway has been implemented through completion of an appropriate questionnaire; discovery of an implemented care pathway triggers drop-out from the control arm. It was estimated that approximately 25% of all CPs have already employed a HF-MPA without yet implementing a HF care pathway.

Practice catchment areas should not overlap, and no other HF care program should be offered regularly to participating patients to reduce contamination bias. Patients in the control group receive usual care concordant with national care guidelines [3] and a trial-specific assessment of primary and secondary outcomes. The trial protocol adheres to the CONSORT extension for cluster-randomized trials [17, 18] and was registered at the German Clinical Trials Register (DRKS00031997).

### Participants

Patients insured under the statutory health insurance and with objectified HF with reduced left ventricular ejection fraction (LVEF ≤ 40%) are identified consecutively in the participating CP or their cooperating GP practices. After initial consultation and trial information by the cardiologist or GP, eligibility confirmation, consent, and enrollment are completed at the CP. In intervention clusters, the HF-MPA assumes responsibility for patient care under delegation. Patients with advanced cognitive impairment or without telephone access were excluded.

### Development of the intervention program

The intervention integrates five synergistic elements. First, evidence-based HF care is implemented in each practice, focusing on patient safety, adherence, and optimization of pharmacotherapy. Second, non-physician staff receive a structured, certified curriculum (HeartNetCare™ [10] provided by the German Society of Cardiology (DGK)) through which they gain qualifications in patient education and symptom monitoring. The training curriculum contents are presented in Supplementary Table S2. Completing the curriculum yields a HF-MPA certificate. Third, an eHealth platform (medPower®; ISO-certified medical product) is deployed, allowing HF-MPAs, cardiologists, and GPs to record, share, and act on clinical data in real time. Fourth, portable telemedical devices (e.g., blood pressure monitors and body weight scales) are provided to enable patient-specific telemonitoring and to ensure timely recognition of clinical deterioration by conducting a preliminary assessment of the collected data based on medically defined and individually set thresholds. Finally, structured communication pathways among cardiologists, GPs, and HF-MPAs ensure coordinated care; HF-MPAs perform scheduled telephone follow-ups, document symptoms and vital signs, and consult the supervising cardiologist or GP when warning signs emerge.

### Role of the HF-MPA

**Fig. 1:**
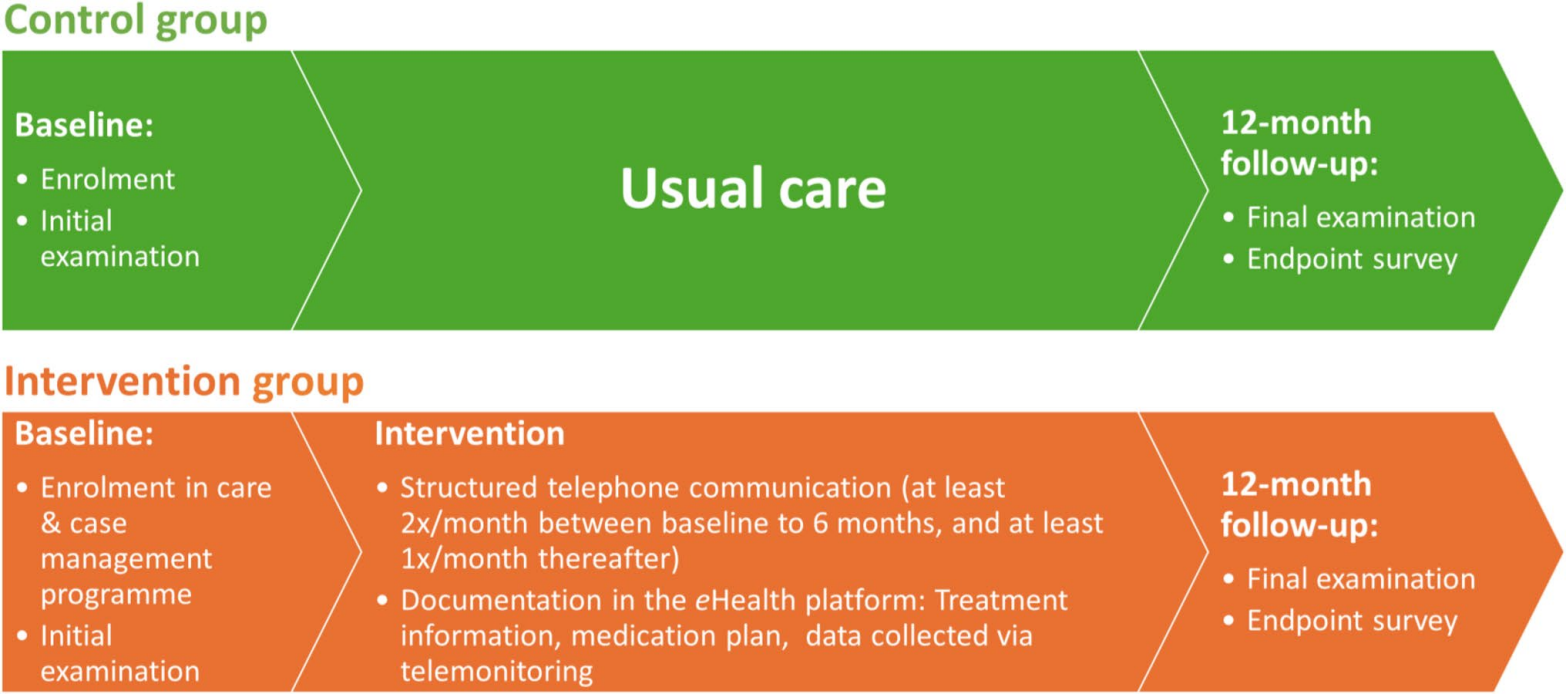
Study flow for control and intervention group

The HF-MPA is employed in the CP and works under the delegation principle. The HF-MPA provides structured contact at program start, conducts scheduled telephone follow-ups and telemonitoring according to patient needs and disease severity, and promptly escalates warning signs. In consultation with the cardiologist, the HF-MPA collaborates with the GP to identify clinical concerns, to implement therapy adjustments and to optimize guideline-concordant medication, when abnormalities are identified. Figure 1 shows the patient pathway and the distribution of patient groups. The HF-MPA is also responsible for documenting care-relevant information using the patient-specific electronic case file of the eHealth-platform. The eHealth-platform is accessible to the patient’s cardiologist, responsible HF-MPA, and the GP, with the contents of the dashboard displayed according to the assigned role and corresponding permissions. The platform is designed to enhance inter-professional and cross-sectoral care coordination and facilitates timely (tele-)monitoring of patients.

### Outcomes

The primary endpoint is the change in HF-specific QoL from baseline to 12 months, measured with the 23-item Kansas City Cardiomyopathy Questionnaire Overall Summary Score (KCCQ-OSS). Its German version has been validated at the Comprehensive Heart Failure Center Würzburg [19]; telephone administration reliability was evaluated prior to the start of the study [20]. The secondary endpoints are presented in Table 1 and include HF-related hospitalization (adjudicated from medical reports) and a health-economic evaluation of first-year intervention costs.

**Table 1:** Secondary endpoints in the HI-PLUS trial

• Composite of unplanned hospitalization due to heart failure (HF) and all-cause mortality
• Components of this composite
• Changes over time in the following parameters:
• Kansas City Cardiomyopathy Questionnaire (KCCQ) Total Symptom Score (KCCQ-TSS)
• KCCQ Clinical Summary Score (KCCQ-CSS)
• Patient Health Questionnaire-9 (PHQ-9)
• Generalized Anxiety Disorder-7 (GAD-7)
• EuroQol-5D-5L
• Montreal Cognitive Assessment (MoCA) test
• New York Heart Association (NYHA) class
• Glomerular filtration rate
• N-terminal pro-B-type natriuretic peptide (NT-proBNP) levels
• Incidental costs in the first year of the intervention through HI-PLUS (health economic project evaluation)
• Acceptance of the new care model
• The previously named quality indicators of the planned Disease Management Program Herzinsuffizienz (DMP-HI) (according to the current planning status of the DMP-HI):
1. Proportion of participants with eGFR and serum electrolytes measured at least every 6 months, relative to all participants with at least 6 months of follow-up 2. Proportion of participants receiving a combination of at least two prognostic agents 3. For participants who were untrained at the time of enrolment: Proportion of participants who attended a recommended training program 4. Proportion of participants with unplanned hospitalization for HF in the previous 12 months, based on all participants with at least 12 months of participation 5. For participants with NYHA Class I-III who were able to exercise: proportion of participants who regularly exercised 6. For participants on Angiotensin-Converting-Enzyme (ACE) inhibitors or angiotensin receptor blockers (ARBs): proportion of participants reaching target dose or maximum tolerated dose 7. For participants taking beta-blockers: proportion of participants reaching the target dose or maximum tolerated dose 8. For participants on mineralocorticoid receptor antagonist (MRA): proportion of participants who reached the target dose or maximum tolerated dose

*HF* heart failure, *KCCQ* Kansas City Cardiomyopathy Questionnaire, *PHQ-9* Patient Health Questionnaire-9, *GAD-7* Generalized Anxiety Disorder-7, *MoCA* Montreal Cognitive Assessment, *NYHA* New York Heart Association, *NT-proBNP* N-terminal pro-B-type natriuretic peptide, *DMP-HI* Disease Management Program Herzinsuffizienz, *eGFR* estimated global filtration rate, *ACE* Angiotensin-Converting-Enzyme, *ARBs* angiotensin receptor blockers, *MRA* mineralocorticoid receptor antagonist

### Data collection

At enrollment, targeted phenotyping captures information on socio-demographic determinants and care situation of patients, as well as HF type and severity, previous history of cardiac disease, comorbid conditions, and current medications. CP teams enter endpoint-relevant information into the study database at baseline and 12 months. Additionally, patients are interviewed by telephone by trained staff to collect endpoints at baseline and 12 months. Quality of care provided to patients is evaluated through the administration of the Patient Assessment of Chronic Illness Care measure (PACIC-5A) at enrollment and the final visit. Furthermore, a qualitative study will be conducted among cardiologists, HF-MPAs, general practitioners, and patients to assess the quality of implementation as well as expectations associated with and acceptance of the innovative care pathway. Study data are managed in REDCap© electronic data capture tools hosted at the University of Würzburg [21, 22]. In the intervention group, care-relevant information is also documented with the eHealth platform. Data monitoring is performed by the Clinical Trial Centre of the University Hospital Würzburg.

### Sample size

The trial is powered to detect a 5-point between-group difference in KCCQ-OSS from baseline to 12 months, a threshold regarded as clinically meaningful and associated with a 10% risk reduction in mortality and HF-related rehospitalization [4, 23–25]. Similar or even greater improvements have been observed in previous therapy optimization studies [26]. The sample size calculation is based on a two-sided *t*-test with degrees of freedom based on the number of clusters, with the significance level set at 5%.

In the INH study, an intraclass correlation coefficient (ICC) of 0.003 was reported for the change in KCCQ-OSS between baseline and 12-month follow-up [10]. Given that the clusters in the INH study comprised cardiology departments within one region, we assume a more conservative ICC of 0.01 for the current trial. For estimating the KCCQ-OSS standard deviation (SD), we refer to the study by Jayaram, which reported an SD of 25.4 points for the change over 6 months [26]. Since the SD remained relatively stable during this period, we assume an SD of 25.4 points after 12 months as well. We also assume a variation in the number of patients per cluster of 0.5. With an ICC of 0.01, an average of 21 HF patients per cluster (*n* = 1050) is needed to detect a significant 5-point difference with 80% power. Considering an expected dropout rate of 20% for patients and 10% for practices, the final sample size is 56 clusters with 1350 patients. Each cardiologist is expected to collaborate with 2–5 GPs, involving up to 280 GPs in total. The sample size was conducted using the software PASS 2020.

### Statistical methods

Analyses will follow the intention-to-treat principle using SAS 9.3 or R. The significance level is defined at 5%. Descriptive analyses of the primary and secondary endpoints will be carried out. The primary analysis will use a univariable linear mixed-effects model with random effects for clusters (CPs) and fixed effects for group (intervention vs control). A multivariable mixed-effects model will secondarily adjust for potential confounders, e.g., age, sex, NYHA functional class, time since diagnosis, care dependency, and living status. A predefined sensitivity analysis will be performed to account for the timing of the anticipated DMP-HI implementation, whose exact start date had not been determined at the time of trial approval. Specifically, a mixed-effects model will be applied to the primary outcome, including both groups and duration of DMP-HI use as fixed effects, while modelling CP as a random effect. If a CP has not participated in the DMP-HI by the end of data collection, duration will be set to zero. If no CP has adopted the DMP-HI 4 weeks before study end, this analysis will be omitted. In addition, a subgroup analysis will evaluate the primary endpoint based on the presence/absence of a trained HF-MPA prior to trial start. Analyses of secondary outcomes will be conducted using univariable and multivariable mixed-effects models. To assess the potential influence of GPs on study outcomes, an additional sensitivity analysis will be run to account for clustering effects at the GP level. This will involve the use of a mixed-effects model with the intervention as a fixed effect and both cardiologists and GPs as random effects.

### Health economic evaluation

A cost-effectiveness analysis will be conducted to estimate the mean differences in costs and effects (e.g., QALYs) between the intervention and control groups. The incremental cost-effectiveness ratio (ICER) will be calculated as the difference in mean costs divided by the difference in mean effects, where applicable. Where one strategy is both more effective and less costly, or vice versa, dominance will be reported instead of an ICER. Effects will be assessed as non-monetary and aggregated appropriately according to the study objectives. Utility values for the calculation of QALYs (Quality-Adjusted Life Years) will be derived from data collected using the European Quality of Life Questionnaire (EuroQoL EQ-5D-5L) using the German value set [27]. Relevant cost parameters will be collected individually to maintain the internal validity of the economic evaluation.

To minimize bias due to recall inaccuracies, resource use data will be collected from the most reliable sources available rather than relying solely on patient self-report. Data on hospitalizations will be extracted from hospital discharge letters, which are systematically reviewed by a clinical expert to identify main diagnoses and relevant procedure codes (OPS), allowing individual-level derivation of Diagnosis-Related Groups (DRGs). The number of visits to cardiologists will be obtained directly from participating cardiologists. Telemonitoring equipment, as an additional resource specific to the intervention, will be continuously recorded via the eHealth platform, whereas additional activities of the HF-MPAs will be documented separately by the HF-MPAs themselves. Information on other healthcare utilization, including GP visits, medications, emergency services, rehabilitation stays, nursing care, and therapeutic services (e.g., physiotherapy, occupational therapy, and speech therapy), will be collected from patients using a questionnaire based on the validated FIMA instrument [28]. Data on care level, living situation, and employment status will also be obtained at baseline and at the end of the observation period.

Costs for non-physician staff, such as HF-MPAs, will be calculated based on existing EBM (Einheitlicher Bewertungsmaßstab) codes and an hourly rate. EBM codes refer to the standardized reimbursement system for outpatient services in Germany, assigning monetary values to specific medical procedures. The patient-related costs of the eHealth platform will be calculated. Drug costs will be estimated using average prices. Furthermore, unit costs for various resources will be derived from published cost estimates [29, 30]. To account for the clustered study design and potential confounders, mixed-effects regression models will be applied, with the intervention group as a fixed effect and clusters (CPs) as random effects. Trial data will be assessed for completeness, and appropriate methods will be applied to handle missing values depending on their extent and underlying mechanism. If data are considered missing at random (MAR), multiple imputation techniques may be employed. Uncertainty in the ICER estimates will be assessed using non-parametric bootstrapping (e.g., 1,000 replications), and results will be presented in cost-effectiveness planes and acceptability curves. Sensitivity analyses will explore the robustness of results to assumptions regarding cost structures, the timing of DMP-HI implementation, and data completeness. All health economic analyses will be conducted using StataSE 17.

### Process evaluation

The implementation and effectiveness of the intervention across clusters will be assessed using a mixed-methods process evaluation guided by the principles outlined by the UK Medical Research Council [31–33]. Key outcomes will focus on patient-level, structural and generalizability factors.

### Pilot feasibility study

A pilot feasibility study was conducted to evaluate key aspects of the trial implementation (Supplementary Material 1).

### Ethical approval

The trial was approved by the Ethics Committee of the University Hospital Würzburg in April 2023 (243/22 me). All recruiting centers obtained local ethics committee approval prior to recruitment.

## Results

The study aims to recruit 1350 patients (2 × 675) in 56 CPs in Germany and follow them for 12 months. The start-up phase started in October 2022; recruitment started in June 2023 and was concluded in June 2025. The last follow-up visit will take place in June 2026. The study will report in September 2026.

**Fig. 2:**
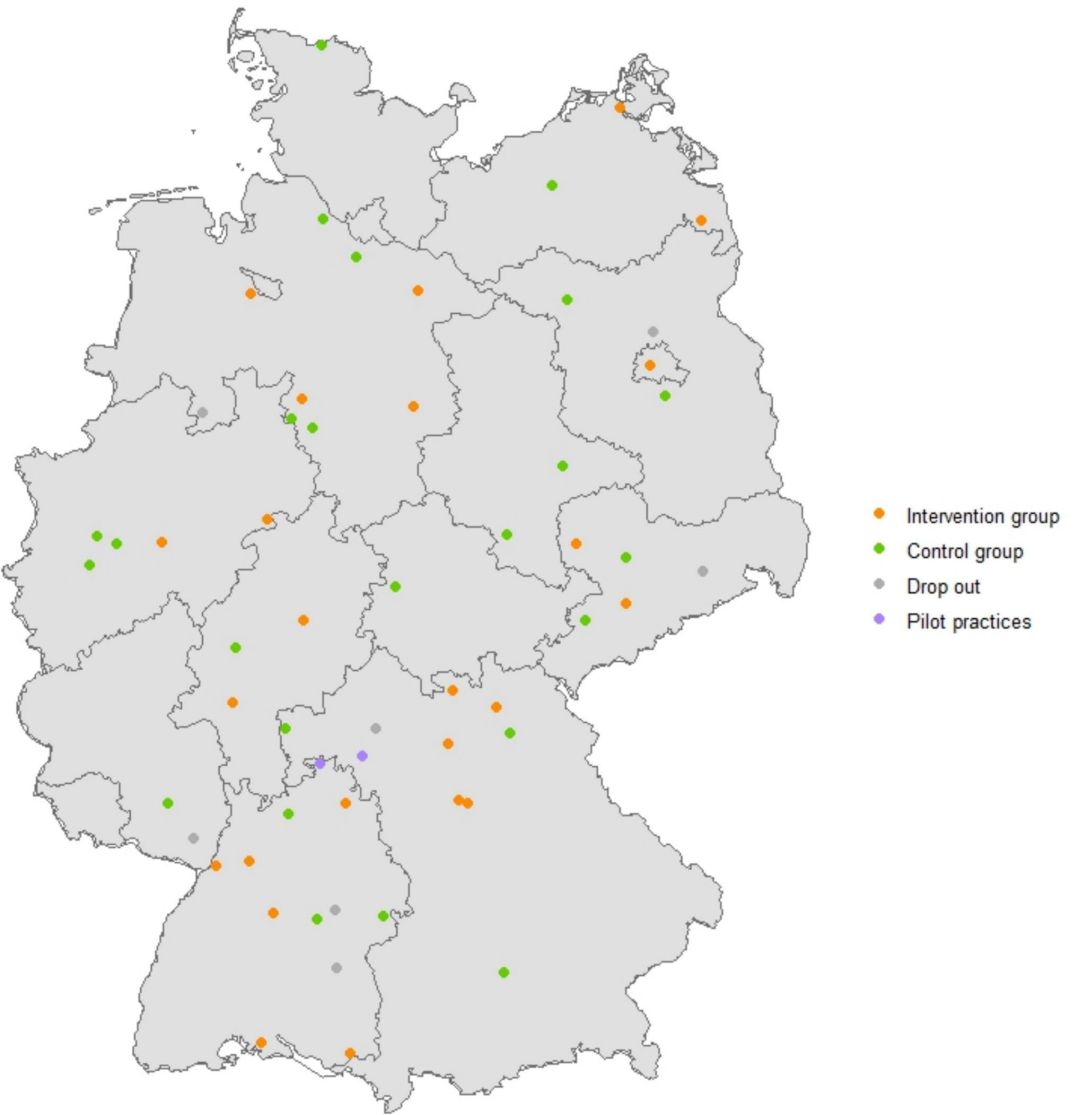
Participating cardiology practices across Germany

Of 1480 cardiologists contacted, 237 cardiologists from 145 CPs expressed interest to participate; 116 CPs received contracts, and 55 CPs (28 intervention and 27 control sites) were ultimately enrolled (Fig. 2). Group allocation takes place after the contract has been signed. Seven CPs (four from the intervention group and three from the control group) withdrew their participation from the trial. None of the CPs had started recruiting patients yet, and the reason for their withdrawal was a shortage of staff or time. All sites received training on recruitment, use of the electronic case report forms (eCRF), and questionnaires. Intervention CPs as well as the certified HF-MPA received additional training on the eHealth platform. The HF-MPA of intervention CPs received specialized HF training according to the curriculum certified by the German Cardiac Society that is based on the evidence-based HeartNetCare™ concept [10].

The CP received an investigator-site file containing the study documents, and the materials were also available online via the cloud. Information about the HI-PLUS trial for patients and potential cooperating physicians is available online at https://www.ukw.de/behandlungszentren/dzhi/forschung/hi-plus/startseite/.

The findings derived from the pilot feasibility study are described in the supplementary information (Supplementary Material 1).

## Discussion

The present manuscript presents the development and feasibility testing of HI-PLUS, an evidence-based structured case and care management program for HF patients in Germany. Delivered by dedicated staff working in delegated care, i.e., HF-MPAs and supported by an eHealth platform, the program aims to strengthen interdisciplinary coordination and implement guideline concordant therapy across care sectors. The pilot feasibility study demonstrated that the program is feasible and acceptable. Lessons from the pilot feasibility study informed refinements for the full-scale trial.

Contemporary guidelines recommend enrolling all HF patients in a multidisciplinary HF management program [2]. However, the evidence regarding improvement in QoL is less consistent than that for reducing hospitalizations. A systematic review of 47 studies involving 10,869 participants evaluated disease management interventions for HF and assessed QoL as a secondary outcome [5]. Although some studies reported QoL benefits, inconsistent reporting precluded definitive conclusions [5]. Evaluating health-related QoL is inherently complex, as it encompasses multiple dimensions of well-being and cannot be adequately captured by clinician-assigned NYHA functional class alone, which may diverge from patient-reported health status [4]. Rather, it requires validated patient-reported outcome measures (PROMs). The KCCQ provides a sensitive, HF-specific assessment of health status and has been validated in multiple settings. Both its 12- and 23-item versions have consistently demonstrated their utility to quantify the impact of HF on disease-specific QoL [23, 34], making the KCCQ well suited as the primary outcome measure for HI-PLUS [35].

Numerous trials investigated patient management and remote patient monitoring (RPM) strategies and reported on mortality and rehospitalizations as outcomes [5, 9–11, 36]. For example, the TIM-HF2 trial, a large randomized multicenter study, demonstrated that structured RPM reduced unplanned cardiovascular hospitalizations and all-cause mortality compared with usual care [11]. Patient education and the individualized use of RPM data were identified as key drivers in preventing major HF events [11]. Similarly, the E-INH trial, which evaluated the long-term effects of a nurse-coordinated DMP, reported reduced mortality at 10 years, suggesting durable benefits when patients are engaged in self-monitoring and adhere to therapeutic recommendations [9, 10]. Notably, KCCQ summary scores improved in both study arms, yet with greater gains in the RPM group that persisted for up to 10 years [9]. In contrast, the SPAN-CHF III trial, which compared tablet-based telemonitoring with an established telephone-based program, found no significant differences in hospitalization days between groups, though it may have been underpowered to detect differences [36]. These heterogeneous results underscore the need to rigorously evaluate the patient-centered benefits of complex interventions and to move beyond hospitalization/mortality as the sole outcome.

Incorporating PROMs as primary endpoints in clinical trials strengthens the patient-centered perspective by capturing complementary dimensions of well-being that are meaningful to patients and by enhancing prognostic accuracy. Despite these advantages, relatively few HF trials use PROMs as primary outcomes, and many clinicians still rely mainly on symptom worsening to guide therapy [37]. Because improving QoL is a central therapeutic goal in HF, strengthening the evidence linking PROMs to interventions is critical. The KCCQ that is validated by the U.S. Food and Drug Administration (FDA) as a regulatory tool [38] is particularly suitable for this purpose. While incorporating PROMs into routine outpatient care can be challenging [37], their feasibility and clinical utility remain essential assets [39]. The shorter, 12-item version of the KCCQ provides a validated and efficient alternative, balancing comprehensiveness with ease of use, thereby facilitating integration into daily practice without unduly burdening physicians or patients [34, 37].

Recruitment of CPs for HI-PLUS has been challenging. Staff shortages, high turnover, and limited resources for trial-related tasks delayed both practice and patient recruitment. To address these barriers, partnerships were formed with professional networks. Furthermore, repeated outreach efforts, including telephone calls and personalized letters, were made to CPs expressing interest. A review of 19 studies on recruiting primary care practices for trials showed that similar strategies were employed in other trials, including in-person meetings or visits by research team members, phone calls, financial incentives, personalized emails or letters, and targeting practices that had participated in previous studies or with which the team had existing connections [40].

Staffing challenges extended beyond recruitment. A qualitative study by Mambrey et al. identified high workload, limited career prospects, and strained interpersonal relationships with supervisors and patients as reasons why MPAs leave the profession [41]. Suggested countermeasures included improved salaries, clearer career pathways, and increased workplace recognition. Additional training programs—such as the HI-PLUS case and care management model—may also increase MPAs’ motivation by expanding responsibilities, strengthening patient relationships, and promoting professional development.

### Feasibility

The pilot feasibility study was designed to evaluate trial processes rather than the intervention itself. Based on feedback from CPs and patients, the study team revised recruitment, consent, and enrollment procedures. Standard Operating Procedures (SOPs) for recruitment, patient consent, and enrollment were revised; expanded patient information materials; and created concise checklists and standard operating procedures to guide study personnel. Study documents were made accessible both by post and via cloud storage, while a concise version of SOPs was created for easy reference. Patient feedback on the telephone interview was positive, with participants reporting appropriate length, clarity, and comprehensibility, so no changes were required. Overall, the pilot feasibility study validated the data collection systems and eHealth platform, demonstrated acceptance among all stakeholders, and provided valuable lessons that have been incorporated into the main trial. Recruitment for the main trial began in June 2023 and the final follow-up is scheduled for completion in the third quarter of 2026.

### Strengths and limitations

A strength of the present trial is the adoption of a structured, evidence-based case and care management program delivered by HF-MPAs trained through a DGK-certified curriculum [10], the use of a validated and FDA-approved PROM (KCCQ) [38] to evaluate patient-reported QoL, and broad geographic representation across Germany. Furthermore, the detailed evaluation of the implementation will provide insights into the introduction of complex interventions into everyday care. Several limitations should also be acknowledged. Recruitment difficulties meant that 55 instead of the targeted 56 CPs could be enrolled, and staff shortages could affect implementation. The intervention is tailored to the German healthcare system, which may limit direct transferability to other contexts; however, delegating aspects of care to trained non-physician staff is a concept that could be adapted internationally. The pilot feasibility study was limited by the small number of patients and did not test the intervention itself. Future pilots of complex interventions might benefit from larger sample sizes and partial implementation to better anticipate operational challenges.

## Conclusion

The HI-PLUS trial has been designed to address recognized deficits in the care of HF patients in Germany. By combining a standardized HF-MPA training with an evidence-based structured care and case-management program embedded in a secure eHealth platform, we aim to foster truly integrated, patient-centered support across the healthcare spectrum. Should the intervention prove beneficial, it may serve not only as a national model for coordinated HF management but also as a blueprint for similar initiatives in other settings, ultimately improving both clinical outcomes and QoL for this vulnerable patient group.

## Supplementary Information

Below is the link to the electronic supplementary material.

Supplementary file1 (DOCX 21.4 KB)

## Data Availability

The individual participant data, after deidentification, that support the findings of the pilot feasibility study, study protocol and informed consent materials can be made available following publication from the corresponding author in its original language to qualified investigators upon reasonable request.
