# Supplementary material for: HI-PLUS: design of a cluster-randomized trial evaluating structured case management with telemonitoring to improve quality of life in heart failure
Source: Clin Res Cardiol. 2026 Apr 27;115(9):1664–74. doi: 10.1007/s00392-026-02904-8 (PMC13457501; doi:10.1007/s00392-026-02904-8)
Supplement: Supplementary file 1 [file 392_2026_2904_MOESM1_ESM.docx]

**Supplementary Information**

**Article title:** HI PLUS: design of a cluster randomised trial evaluating structured case management with telemonitoring to improve quality of life in heart failure

**Journal name:** Clinical Research in Cardiology

**Author names:** Martha Schutzmeier^1,6^, Viktoria Rücker^1^, Jonas Widmann^1^, Peer Papior^2^, Lisa Kimmelmann^2^, Fabian Kerwagen^2^, Lorenz Heil^3^, Helena Manger^3^, Andrea Szczesny^3^, Yannick Maaser^4^, Bettina Zippel-Schultz^4^, Thomas Helms^4^, Peter Heuschmann^1,5,6^, Stefan Störk^2*^

**Affiliations:**

1. University of Würzburg, Institute for Clinical Epidemiology and Biometry, Würzburg, Germany

2. University Hospital of Würzburg, Comprehensive Heart Failure Center Würzburg, Würzburg, Germany

3. University of Würzburg, Department of Business Management, Würzburg, Germany

4. German Foundation for the Chronically Ill, Fürth, Germany

5. University Hospital of Würzburg, Clinical Trial Center, Würzburg, Germany

6. University Hospital Würzburg, Institute of medical Data Science, Würzburg, Germany

**E-mail address of the corresponding author:** Stoerk_S@ukw.de

**Supplementary Material 1**

**Methods of the pilot feasibility study**

A pilot feasibility study was conducted to evaluate key aspects of trial implementation. The objectives were to assess the time need to find patients and the recruitment approach, test electronic case report forms (eCRFs) and electronic patient files within the eHealth platform, evaluate the comprehensibility and length of the patient questionnaires, estimate the duration of the baseline assessment, and gather feedback from the cardiological practice (CP) regarding patient willingness to participate. During this phase, the baseline assessment was tested, which included a physical examination, a consultation with the cardiologist and a standardized telephone survey administered by trained staff. The study database and the eHealth platform were also piloted. At the end of the pilot feasibility study, both the participating CP and the study participants completed a feedback questionnaire.

**Results of the pilot feasibility study**

Two CPs participated in the pilot feasibility study between May and July 2023; each recruited 2 patients (3 men aged 52, 69, and 80 years, and 1 woman aged 73 years). All screened patients consented to participate. The process of informing patients, conducting the baseline assessment, and entering data required approximately 60 minutes per patient. The duration of the telephone survey averaged 40 ± 8 minutes. Feedback from CPs prompted refinements to the Standard Operating Procedures (SOPs) for recruitment, consent procedures and standard operating protocols. Patients reported high satisfaction and good comprehensibility of the telephone survey.

**Supplementary** **Table S1**: Trial organization

**Project Leader and Coordinating Investigator**

Stefan Störk, Universitätsklinikum Würzburg – Deutsches Zentrum für Herzinsuffizienz, Würzburg

**Evaluating institution**

Institute for Clinical Epidemiology and Biometry - University of Würzburg, Würzburg (Prof. Dr. Peter Heuschmann)

**Scientific Advisory Board**

Aktionsbündnis Patientensicherheit e.V. (APS), Bundesverband Niedergelassener Kardiologen (Dr. Norbert Smetak), Defibrillator (ICD) Deutschland e.V. (Matthias Kollmar), Deutsche Gesellschaft für Kardiologie, AG 10 Chronische Herzinsuffizienz (Prof. Dr. Philip Raake), Deutscher Hausärzteverband e.V., Prof. Dr. Friedrich Köhler (Charité – Universitätsmedizin Berlin), Prof. Dr. Michael Böhm and Prof. Dr. Ingrid Kindermann (Universitätsklinikum des Saarlandes)

**Advisory Board**

Abbott Medical GmbH (Stefan Döpel), BNK Saarland (Dr. Jörg Schmidt), Forcare GmbH, Kassenärztliche Vereinigung Saarland (Wolfgang Herian), Ministerium für Soziales, Gesundheit, Frauen und Familie des Saarlandes, Novartis Pharma GmbH (Dr. Herdis Bender), Vifor Pharma GmbH (Dr. Beate Stremmel), WZAT Westdeutsches Zentrum für angewandte Telemedizin GmbH, pathways (Dr. Holger Friedrich)

**Biostatistics**

Viktoria Rücker, Institute for Clinical Epidemiology and Biometry - University of Würzburg, Würzburg

**Funding**

The HI-PLUS trial is supported by the German Innovation Fund of the Federal Joint Committee (“Innovationsfonds des Gemeinsamen Bundesausschusses”, grant number 01NVF19029).

**Event Adjudication Committee**

An Endpoint Adjudication Committee (EAC) has been established that will be blinded to study group assignment. The EAC will review and adjudicate all hospitalisations using the prospectively defined criteria outlined in the EAC charter.

**Clinical Research Organization**

Data monitoring is performed by the Clinical Trial Centre of the University Hospital Würzburg.

**Lead Ethics Committee**

The study was approved in April 2023 by the central Ethics Committee at the University Hospital Würzburg (registry number 243/22-me).

**Trial Registration**

The trial was registered under the number DRKS00031997 at the German Clinical Trials Register on 31 May 2023. Enrollment of the main trial started in June 2023.

**Supplementary** **Table S2**: Overview of the training curriculum

**Content structure:**

**Module 1:**

- Significance and epidemiology of heart failure

- Definition and aetiology

**Module 2:**

- Diagnosis of heart failure

**Module 3:**

- Pharmacology

- Non-pharmacological therapy options

- Surgical methods/resynchronisation therapy

**Module 4:**

- Comorbidities and their management in heart failure

**Module 5:**

- Telephone monitoring and telemonitoring

**Module 6:**

- Structured care in the network

- Quality assurance

**Teaching structure:**

- 32 hours of theoretical lessons (delivered through seminars and small-group activities)

The curriculum concludes with a written knowledge assessment.

**Eligibility criteria:**

- Qualification as a medical assistant

- Minimum of three years’ experience in outpatient cardiology care

- Active role as a medical tutor in a general practice setting
